# Genomic Mutations of the STAT5 Transcription Factor Are Associated with Human Cancer and Immune Diseases

**DOI:** 10.3390/ijms231911297

**Published:** 2022-09-25

**Authors:** Uijin Kim, Ha Youn Shin

**Affiliations:** Department of Biomedical Science and Engineering, Konkuk University, Seoul 05029, Korea

**Keywords:** STAT5, transcriptional regulation, genomic mutations, human diseases

## Abstract

Signal transducer and activation of transcription 5 (STAT5) is a key transcription factor that regulates various biological processes in mammalian development. Aberrant regulation of STAT5 has also been causally linked to many diseases, including cancers and immune-related diseases. Although persistent activation of STAT5 due to dysregulation of the signaling cascade has been reported to be associated with the progression of solid tumors and leukemia, various genomic mutations of STAT5 have also been found to cause a wide range of diseases. The present review comprehensively summarizes results of recent studies evaluating the intrinsic function of STAT5 and the link between STAT5 mutations and human diseases. This review also describes the types of disease models useful for investigating the mechanism underlying STAT5-driven disease progression. These findings provide basic knowledge for understanding the regulatory mechanisms of STAT5 and the progression of various diseases resulting from aberrant regulation of STAT5. Moreover, this review may provide insights needed to create optimal disease models that reflect human disease associated STAT5 mutations and to design gene therapies to correct STAT5 mutations.

## 1. Introduction

The signal transducer and activator of transcription (STAT) proteins are a family of transcription factors that mediate diverse biological processes, including immune responses, cell proliferation, and differentiation. The first two STAT proteins, STAT1 and STAT2, were identified in the interferon system as cytoplasmic transcription factors [1]. STAT1 and STAT2 are activated by Janus kinases (JAKs) and bind to the specific target sequence of a promoter for interferon-alpha (IFN-α) and IFN-gamma (IFN-γ) gene induction. Subsequently, additional STAT family members with distinct biological functions were identified. At present, the STAT family consists of seven members (STAT1, STAT2, STAT3, STAT4, STAT5A, STAT5B, and STAT6) with similar molecular weights ranging from 75 to 95 kDa [2]. The STAT5A protein was initially identified as a mammary gland factor that regulates genes encoding milk proteins in response to lactogenic hormones [3,4]. A structurally related isoform, STAT5B, was subsequently identified [5,6,7]. The two isoforms, STAT5A and STAT5B, collectively known as STAT5, are activated by many cytokines and growth factors, including prolactin (PRL), growth hormone (GH), interleukin 2 (IL-2), IL-3, and erythropoietin (EPO), with STAT5A and STAT5B showing distinct and partially redundant biological functions [8,9,10]. Persistent STAT5 activity has also been causally linked to solid tumors and leukemia [11,12,13]. The present review focuses on the unique features of STAT5 and the genomic STAT5 mutations associated with various diseases. This review also describes current disease models useful for studying the role of STAT5 in disease progression and proposes strategies for treating STAT5 mutation-driven diseases.

### 1.1. Genomic Structure and Functional Domains of STAT5

**Figure 1:**
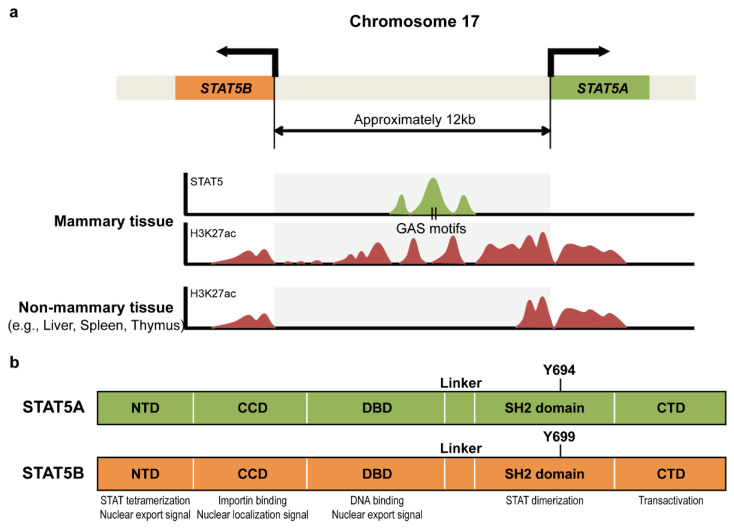
Schematic diagrams of the STAT5 genomic locus and its functional domains. (**a**) Genomic location of genes encoding STAT5A and STAT5B. The transcription start sites of *STAT5A* and *STAT5B* are oriented in opposite directions within a ~12 kb sequence on human chromosome 17. ChIP-seq analyses revealed that the STAT5-bound enhancer is only found in the intergenic region between the *STAT5A* and *STAT5B* genes in mammary tissue and is essential for autoregulation of STAT5 transcription. (**b**) Functional domains of STAT5A and STAT5B. The NTD is required for STAT5 tetramerization and contains a Crm1 exportin-mediated nuclear export signal. The CCD contains a nuclear localization signal and binds to importin proteins. The DBD binds to the STAT5 binding motif of the target promoter and contains a Crm1-independent nuclear export signal. The SH2 domain is important for STAT5 homo- or heterodimerization and the CTD functions in transactivation. Phosphorylation of a specific tyrosine residue (Y694 of STAT5A and Y699 of STAT5B) is essential for STAT5 activation by JAK. Abbreviations: NTD, N-terminal domain; CCD, coiled-coil domain; DBD, DNA-binding domain; SH domain, Src homology 2 domain; CTD, C-terminal transactivation domain.

STAT5A and STAT5B are encoded by two distinct genes positioned in a head-to-head orientation within ~10 kb on human chromosome 17 and mouse chromosome 13 [10,14] (Figure 1a). ChIP-sequencing (ChIP-seq) profiling and clustered regularly interspaced short palindromic repeats (CRISPR)-Cas9-targeted mice have revealed that transcription of the STAT5 gene is auto-regulated by a STAT5-driven enhancer in mammary tissue [15]. Although STAT5A and STAT5B proteins are encoded by separate genes, they are 90% identical at the amino acid level and their functional domains are similar to those of other STAT family members [16]. The STAT protein consists of an N-terminal domain, a coiled-coil domain, a DNA binding domain, a linker region, a Src homology 2 (SH2) domain, and a C-terminal transactivation domain. STAT5A and STAT5B contain 794 and 787 amino acids, respectively, and the arrangement of their functional domains is highly conserved, with slight differences in their C-terminal domains [7] (Figure 1b). The N-terminal domain is essential for STAT5 tetramerization and contains a nuclear export signal, whereas the coiled-coil domain contains an unconventional nuclear localization signal [17,18]. The DNA-binding domain binds to the STAT5 binding motif of the target promoter and contains a nuclear export signal, and the SH2 domain is required for STAT5 dimerization. Phosphorylation of a specific tyrosine (Y) residue, Y694 in STAT5A and Y699 in STAT5B, is essential for activation of these proteins by JAK. The C-terminal transactivation domain is responsible for the binding of STAT5 to DNA [19].

### 1.2. Activation Mechanism and Biological Functions of STAT5

**Figure 2:**
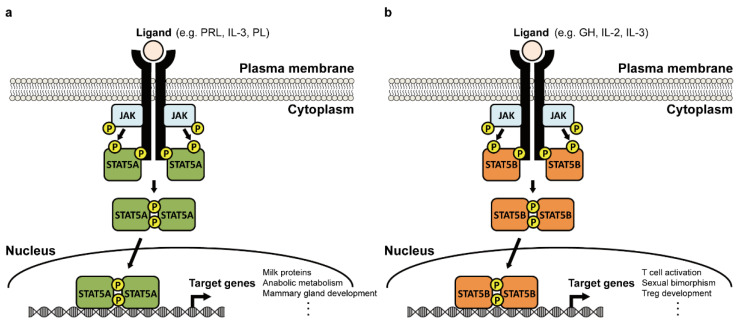
STAT5 activation mechanism. (**a**) STAT5A is activated by Janus kinases (JAK) in response to prolactin (PRL), interleukin-3 (IL-3), and placental lactogen (PL) to induce the expression of genes involved in mammary gland development and immune responses. (**b**) STAT5B is activated by JAK in response to growth hormone (GH), IL-2, and IL-3 and induces genes involved in body growth and immune responses.

STAT5 is a latent cytoplasmic transcription factor that can be activated in response to a variety of cytokines, hormones, and growth factors [20], resulting in its translocation into the nucleus (Figure 2). Once ligands bind to the cell surface receptors, the associated JAKs phosphorylate the specific tyrosine residues (Y694 of STAT5A or Y699 of STAT5B) and activated STAT5 forms homo- or heterodimers through reciprocal SH2 domains. STAT5 dimers then translocate to the nucleus and bind to a specific sequence called the Interferon Gamma Activated Sequence (GAS motif, TTCNNNGAA) in the promoter of responsive genes. Phosphorylation of specific serine residues (S725 and S779) in STAT5A was also found to be involved in hematopoietic transformation [21,22]. Unphosphorylated STAT5 continuously shuttles between the nucleus and cytoplasm [18], although its specific function remains to be determined.

Studies in STAT5 null mice revealed that STAT5A and STAT5B have partially redundant and partially distinct functions [16]. Loss of STAT5A in mice impaired mammary gland development and alveolar differentiation during pregnancy [8], whereas mice lacking STAT5B exhibited defective body growth [23]. Inactivation of STAT5A or STAT5B is partially compensated by the other, whereas the absence of both induced perinatal lethality [9,24]. STAT5A is predominantly expressed in the mammary glands, whereas STAT5B is more abundant in the liver, muscle, and immune cells [15]. Because of differences in their levels of STAT5 expression across cell types, STAT5A and STAT5B are sensitive to distinct cytokines. STAT5A is predominantly stimulated by PRL and induces genes involved in alveolar differentiation and milk secretion (Figure 2a). STAT5B activated by GH enhances body growth and liver gene expression, whereas STAT5B activated by IL-2 or IL-3 enhances immune responses (Figure 2b).

## 2. Dysregulation of STAT5 in Human Diseases

Persistent activation of STAT5 has been causally linked to tumorigenesis, including breast cancer, colorectal cancer, and hematological malignancies [25]. Human leukemia patients have shown continual activation of STAT5 by JAK mutations, BCL-ABL chimeric oncoproteins, TEL-JAK2 fusion proteins, or FLT-ITD mutations [12,26,27,28,29,30]. Recent advances in genome sequencing technology have shown that genomic mutations of STAT5 result in the aberrant regulation of diverse signaling pathways and cause various diseases [31,32,33,34].

### 2.1. Diseases Caused by Dysregulation of the STAT5 Signaling Pathways

STAT5 is a signal transducer primarily activated by members of the JAK family, a group of non-receptor tyrosine kinases that transduce cytokine signaling. To date, four members of the JAK family have been identified: JAK1, JAK2, JAK3, and tyrosine kinase 2 (TYK2). These proteins share similar functional domains, including an amino-terminal FERM homology domain, a SH2 domain, a pseudokinase domain called JAK homology 2 (JH2), and a tyrosine kinase domain called JH1. Mutations in JAKs have been found to induce the persistent activation of STAT5, resulting in solid cancers and leukemia (Table 1). The missense mutation A634D in JAK1 enhances STAT5 phosphorylation and is associated with T cell- and B cell-acute lymphoblastic leukemia [35]. The gain of function mutation S703I in JAK1 also results in the constitutive upregulation of STAT5 phosphorylation in patients with autoinflammatory diseases [36]. In contrast, exome sequencing analysis identified a germline mutation in JAKl, P733L, which reduced STAT5 phosphorylation in immunodeficient patients with early metastatic bladder carcinoma [37]. The somatic mutation V671F of JAK2 was found to constitutively activate JAK2 activity and to be highly associated with myeloproliferative cancer [12]. Germline mutation E846D in JAK2 causes the prolonged Epo-dependent activation of STAT5 and contributes to hereditary erythrocytosis [38]. The missense mutation M511I in JAK3 results in the hyper-phosphorylation of STAT5 and is associated with T-cell prolymphocytic leukemia [39].

The constitutively active tyrosine kinase BCR-ABL hybrid oncogene is highly prevalent in patients with chronic myeloid leukemia [40]. This BCR-ABL fusion gene is generated by chromosomal translocation between chromosomes 9 and 22. STAT5 activity is increased in BCR-ABL^+^ leukemia [41], with the production of reactive oxygen species (ROS) by STAT5 exacerbating the progressive stage of the disease [42]. Several missense mutations in BCL-ABL, including E255K, D276G, F359V, and F317L, have been found to induce resistance to treatment and worsen patient prognosis [43].

### 2.2. Diseases Associated with STAT5 Genomic Mutations

Genetic mutations in STAT5 have been associated with various diseases (Table 2). Genomic mutations, including point mutations, insertions, and deletions, have been identified in both the coding and noncoding regions of STAT5. A single nucleotide polymorphism (SNP) in STAT5 was found to be highly associated with chronic inflammatory diseases, such as Crohn’s disease and atopic dermatitis [31,32]. STAT5 rs16967637 was found to be associated with dysregulation of T cell differentiation by altering the numbers of T-helper 17 (Th 17) and T-regulatory (Treg) cells that are strongly associated with Crohn’s disease [31]. STAT5A SNP rs16967637 was also found to exacerbate the severity of atopic dermatitis in African Americans, whereas STAT5B SNP rs9900213 was found to increase the risk of atopic dermatitis in European Americans [32]. Case studies have shown that STAT5A SNP rs7217728 and STAT5B SNP rs6503691 and rs7218653 are highly associated with colon cancer [47]. 

Several missense mutations in STAT5 were found to promote cytokine-independent cell proliferation in solid tumors and leukemias. Integrated exome sequencing and mRNA sequencing analyses revealed that E269Q, a missense point mutation in the coiled-coil domain of STAT5A, was associated with head and neck squamous cell carcinoma (Cosmic database COSV61805753) [48]. Introduction of a single point mutation N642H near the phosphotyrosine-binding site in the SH2 domain of STAT5A was found to induce the constitutive activity of STAT5 [49,50], promoting the GM-CSF-independent growth of the TF-1 human leukemic cell line and IL-6-dependent differentiation of the M1 murine leukemic cell line. Indeed, STAT5 N642H mutations have been identified with high frequency in patients with T-cell prolymphocytic leukemia [51]. The STAT5B N642H mutation is also frequently detected in pediatric T-cell acute lymphoblastic leukemia and has been associated with an increased risk of recurrence [52]. Whole genome sequencing analysis showed that the STAT5B N642H mutation was associated with aggressive mature T-cell leukemia [33,34], and RNA-sequencing analysis found that the recurrent STAT5B N642H mutation was present in myeloid neoplasms with eosinophilia [53]. Integrated genomic analysis identified another missense mutation Y665F in STAT5B that induces constitutive phosphorylation of mutant protein in patients with T-cell acute lymphoblastic leukemia and T-cell prolymphocytic leukemia [54].

In addition to point mutations, insertions and deletions are also frequently found in STAT5 mutation-driven diseases. An insertion in the DNA binding domain of STAT5A, the Q368Pfs*9 mutation, has been associated with colorectal cancer (Cosmic database COSV61805871) [55]. In STAT5B, a deletion mutation (K583Nfs*16) in the DNA binding domain that resulted in a frameshift was found to correlate with colorectal cancer and breast cancer (Cosmic database COSV53180593) [56].

## 3. Mutation Models Developed to Study the Role of STAT5 in Diseases

The role of STAT5 in mammalian development and disease has been investigated by the introduction of different types of mutations into cells and/or animal models. Many mutations in STAT5 or STAT5-associated tyrosine kinases have been generated by the conventional Cre-Lox recombination system, resulting in full or partial gene deletions [9,24]. Recent advances in genome editing technology, such as the CRISPR-Cas9 system, have allowed the generation of target specific mutations in a time and cost-effective manner (Table 3). The CRISPR-Cas9 system was first detected in bacterial immune systems and was further engineered into a two component system, a single guide RNA (sgRNA) and a Cas9 endonuclease, for genome editing [60]. Once the gRNA binds to the target sequence, the Cas9 generates a double-stranded break 3 bp upstream of the protospacer adjacent motif (PAM, NGG sequence). The cleaved region is further degraded by cellular exonucleases and repaired by non-homologous end joining, which can result in the deletion of several nucleotides from the target region. This newly developed genome editing technology enables target-specific mutations in various organisms [61,62,63].

### 3.1. Disease Models with STAT5 Genomic Mutations

Recent advances in genome editing technologies have enabled researchers to utilize the CRISPR-Cas9 system to generate disease models with STAT5 mutations. For example, STAT5A, STAT5B, and STAT5A/B deletion mutations were generated in a T lymphoblastoid cell line using the CRISPR-Cas9 system to investigate the role of STAT5 in glucocorticoid (GC) resistant T cell-acute lymphoblastic leukemia [64]. IL-7 was found to promote resistance to GC therapy in patients with T cell-acute lymphoblastic leukemia, suggesting that this disease could be treated by inhibiting the IL-7R/JAK/STAT5 pathway. CRISPR-Cas9-mediated STAT5A/B deletions were induced in a neuroblastoma cell line to determine the correlation of neuroprotective activity with the IFN-β/STAT5 pathway in neurodegenerative diseases such as Parkinson’s disease [65]. Results of this study suggested that STAT5 is essential for IFN-β-mediated mitochondrial homeostasis and that the IFN-β/STAT5 molecular cascade could be a potential therapeutic target for neurodegenerative diseases. A STAT5B deletion mutation was generated in a malignant human B cell line using the CRISPR-Cas9 system to elucidate the role of STAT5 in the differentiation of B cell subsets [66]. These results identified the mechanisms regulating STAT5B in IL-21-dependent human B-cell differentiation and the correlation of STAT5B deficiency with dysfunctional humoral immunity in autoimmune diseases.

Prior to the widespread use of the CRISPR-Cas9 genome editing technology, sequence-specific mutagenesis was introduced with retroviral vector systems, based on the ability of retroviruses to stably integrate into the chromosomes of dividing target cells [68]. The role of STAT5 mutations in disease was therefore investigated by using retroviral vectors to introduce STAT5 missense mutations into target cells. In addition to the persistent STAT5 phosphorylation, STAT5 frequently shows post-translational modifications, such as glycosylation at T92, in many human cancer cells. Mice with B cells carrying the STAT5 T92A mutation were generated using a retroviral vector system [67], with this mutation resulting in decreased STAT5 phosphorylation, transactivation potential, and oncogenic transformation capacity in mice, suggesting that T92 could be a potential therapeutic target. A constitutively active form of STAT5A with H299R and S711F mutations was also introduced into a pro B cell line through PCR-driven mutagenesis followed by retroviral transduction [68]. This mutant cell line was found to constitutively express an active form of STAT5 and grow independent of IL-3 stimulation, making it useful for studying the role of STAT5 in leukemogenesis. The serine 726 and 780 residues were recently shown to be constitutively phosphorylated in breast cancer cell lines and patient tumor samples [69]. To investigate the role of serine phosphorylation of STAT5 in breast cancer development, STAT5 was knocked down with shRNA and phospho-deficient mutations (S726A and S780A) were introduced into the MCF7 breast cancer cell line through lentiviral transduction. Loss of serine phosphorylation reduced the PRL-induced proliferation of MCF-7 cells independent of tyrosine phosphorylation, indicating that these specific serine residues may be molecular targets for clinical intervention.

### 3.2. Other Mutation Models Useful to Study the Role of STAT5 in Tumorigenesis

The CRISPR-Cas9 genome editing technique has also been utilized to create models evaluating the role of STAT signaling pathways in disease. For example, the mechanism of JAK1/STAT3 regulation in tumorigenesis was assessed by using the CRISPR-Cas9 system to introduce a JAK1 deletion mutation into a mouse fibroblast cell line overexpressing the ubiquitin-specific protease 6 (USP6) oncogene [70]. Similarly, to investigate JAK3 function in autoimmune diseases, a CRISPR-Cas9-mediated JAK3 deletion was generated in an induced pluripotent stem cell (iPSC) line derived from a patient with severe combined immunodeficiency (SCID) [71]. CRISPR-Cas9-directed JAK3 correction restored the normal T cell development. In contrast, acute myeloid leukemia cell lines carrying the most prevalent JAK mutation, V671F, were screened with the amplification refractory mutation system (ARMS) that detects single nucleotide changes or small deletions [72]. These JAK-deficient or mutant cell lines will be useful to study the role of STAT5 in tumorigenesis, as multiple studies have reported that the aberrant JAK/STAT5 pathway is involved in cancer progression.

A drug resistant leukemia cell line was generated by introducing the T315I mutation into the BCR-ABL^+^ leukemia cell line using the CRISPR-Cas9 system [73]. This cell line showed resistance to the tyrosine kinase inhibitor imatinib, suggesting that this BCR-ABL mutant cell line will be useful to study the correlation of BCR-ABL oncogenes and STAT5 in the development of leukemia.

## 4. Discussion

STAT5 plays an important role in regulating mammalian development and disease progression. Dysregulation of STAT5 has been found to be associated with many human diseases [26,29,30,40]. As accumulating evidence has shown that constitutive activation of STAT5 by abnormal JAK activity is a major cause of cancer and hematological malignancies, several JAK inhibitors have been used to treat STAT5-associated diseases [74,75]. For example, Ruxolitinib (Jakafi^®^, Incyte Corp., Wilmington, DE, USA), a JAK inhibitor that targets both JAK1 and JAK2, was first approved by the United States Food and Drug Administration (USFDA) in 2011 for the treatment of myeloid disorders [76,77,78,79]. Tofacitinib (Xeljanz^®^, Pfizer, New York, NY, USA) is another JAK inhibitor that selectively targets JAK3, which was approved by the USFDA in 2012 for the treatment of rheumatoid arthritis and more recently for the treatment of ulcerative colitis [78,80,81]. Pacritinib, a JAK2 and FTL3 inhibitor, is currently in phase 2 clinical trials for prostate cancer (Clinical trial number: NCT04635059). Several inhibitors that directly target STAT5 have also been developed. Pimozide is a psychotropic drug that inhibits STAT5 tyrosine phosphorylation and induces cell death in chronic myeloid leukemia [44]. IST5-002 is a small molecule inhibitor that prevents both JAK2 and BCR-ABL-mediated phosphorylation of STAT5 and induces apoptosis in prostate cancer and chronic myeloid leukemia [82]. AC-4-130 is a STAT5 SH2 domain inhibitor that directly inhibits STAT5 activation, dimerization, and translocation in acute myeloid leukemia [83].

Although persistent activation of STAT5 by aberrant kinase activity has been found to promote tumorigenesis, little is known about the correlations between disease and genomic mutations in STAT5 or in its regulatory regions. Recent advances in next-generation sequencing technology have revealed hitherto unknown STAT5 genomic mutations in human disease (Table 2). Integration of whole genome sequencing and transcriptome analysis of clinical samples will be useful for identifying disease-associated STAT5 genetic mutations at the whole-genome level. However, studies of cells and animal models with STAT5 mutations are essential to investigate the genuine function of disease-associated STAT5 mutations. Although the conventional Cre-Lox recombination system has been widely used to study the biological functions of many molecules, it is technically difficult, takes a long time to obtain mutations, and has a low mutation rate. The recently developed CRISPR-Cas system has provided an unprecedented opportunity to generate target-specific mutations in a wide range of organisms in a time- and cost-efficient manner. In addition, the simultaneous introduction of gRNAs targeting multiple sites can induce the deletion of multiple sites in a relatively short time [84].

To date, the CRISPR-Cas9 system has only been used to generate STAT5 deletion mutants, whereas retroviral transduction systems have been mainly used for the replacement or insertion of site-specific sequences (Table 3). Although CRISPR-Cas9-mediated homologous recombination leads to low insertional mutation rates, recently developed base-editing and prime-editing systems have enabled the precise introduction of point mutations or multiple nucleotide exchanges with high frequency [85,86]. Base-editing can exchange single nucleotides from C to T or G to A using a catalytically inactive Cas9 linked to cytidine deaminase or adenosine deaminase, respectively [85,87]. Prime editing can even replace short nucleotides using Cas9 nickase fused with reverse transcriptase [86]. These techniques can introduce disease associated STAT5 SNPs, missense mutations, or insertional mutations into cells and animal models. STAT5 mutant models can help evaluate the direct impact of STAT5 mutations on disease progression and enable understanding of the mechanisms underlying STAT5-induced disease development. Furthermore, genome editing techniques such as CRISPR-Cas9, base-editing, and prime-editing can be exploited for gene therapy of STAT5-associated diseases. Although these techniques require improvement to avoid unexpected off-target events and more efficient intracellular delivery systems are needed, they have clearly revolutionized the field of genome editing. The efficiency of intracellular delivery of the CRISPR-Cas system have been improved by developing a smaller version of Cas9 endonuclease, such as CjCas, to fit the genome capacity of an adeno-associated viral vector, or by utilizing a baculoviral vector with a packaging capacity over 100 kb [88,89]. To further improve the mutation rate, a single plasmid expressing gRNA cassettes and Cas9 nuclease has been developed as an alternative to introducing gRNA and Cas9 into cells separately [90]. These strategies could facilitate the development of clinical therapeutics to treat STAT5 genetic mutation-driven diseases.

## Figures and Tables

**Table 1:** Mutations in tyrosine kinases associated with STAT5-driven disease.

Gene	Mutation	Region	Disease	Reference
*JAK1*	A634D	Pseudokinase	Acute lymphoblastic leukemia	[35]
	S703I	Pseudokinase	Autoimmune disease	[36]
	P733L	Pseudokinase	Bladder carcinoma	[37]
*JAK2*	V617F	Pseudokinase	Myeloproliferative disorders	[26,44,45]
	E846D	Linker	Erythrocytosis	[38,46]
*JAK3*	M511I	Linker	Leukemia	[39]
*BCR-ABL1*	E255K	P-Loop	Leukemia	[40,42,43]
	D276G	Linker	Leukemia	[42]
	F359V	Linker	Leukemia	[40,42,43]
	F317L	Linker	Leukemia	[40,42,43]

**Table 2:** Types of STAT5 genomic mutations associated with disease.

Gene	Mutation	Region	Disease	Reference
*STAT5A*	SNP (rs16967637)	Intron	Crohn’s disease	[31]
			Atopic dermatitis	[32]
	SNP (rs7217728)	Intron	Colon cancer	[47]
	Missense (E269Q)	Coiled-coil domain	Upper aerodigestive tract cancer	[48]
	Missense (N642H)	SH2 domain	Leukemia	[49,50,51]
*STAT5B*	SNP (rs9900213)	Intron	Atopic dermatitis	[32]
	SNP (rs6503691, rs7218653)	Intron	Colon cancer	[47,57]
	Missense (N642H)	SH2 domain	Leukemia	[52,53,58,59]
	Missense (Y665F)	SH2 domain	Leukemia	[33,54,59]
*STAT5A*	Frameshift (Q368Pfs*9)	DNA binding domain	Colorectal cancer	[55]
*STAT5B*	Frameshift (K583Nfs*16)	SH2 domain	Colorectal cancer, Breast cancer	[56]

**Table 3:** Mutation models useful to study the role of STAT5 in diseases.

Mutation	Gene Editing Method	Species and Cell Type	Disease	Reference
*STAT5A/B* deletion	CRISPR-Cas9	Human T lymphoblast cell	Leukemia	[64]
	CRISPR-Cas9	Mouse neuroblast cell	Neurodegeneration	[65]
*STAT5B* deletion	CRISPR-Cas9	Human B cell	Autoimmune disease	[66]
*STAT5A/B* T92A	Viral transduction	Mouse bone marrow cell	Oncogenesis	[67]
*STAT5A* H299R, S711F	Viral transduction	Mouse pro-B cell	Leukemia	[68]
*STAT5A* S726A, S780A	Viral transduction	Human breast cancer cell	Breast cancer	[69]
*JAK1* deletion	CRISPR-Cas9	Mouse fibroblast cell	Tumorigenesis	[70]
*JAK3* deletion	CRISPR-Cas9	SCID patient-derived iPSC	Autoimmune disease	[71]
*JAK2* V617F	Amplification refractory mutation system (ARMS)	Human B cell	Leukemia	[72]
*BCR-ABL1* T315I	CRISPR/Cas9	Human BCR-ABL^+^ leukemia cell	Leukemia	[73]

## Data Availability

Not applicable.
